# Prognostic significance of Rab27 expression in solid cancer: a systematic review and meta-analysis

**DOI:** 10.1038/s41598-020-71104-9

**Published:** 2020-08-24

**Authors:** Hyun Min Koh, Bo Gun Jang, Dong Chul Kim

**Affiliations:** 1 Department of Pathology, Gyeongsang National University Changwon Hospital, Changwon, South Korea; 2 Department of Pathology, Jeju National University School of Medicine, Jeju, South Korea; 3 Department of Pathology, Jeju National University Hospital, Jeju, South Korea; 4 Department of Pathology, Gyeongsang National University School of Medicine, 79 Gangnam-ro, Jinju, 52727 South Korea; 5 Department of Pathology, Gyeongsang National University Hospital, Jinju, South Korea; 6 Gyeongsang Institute of Health Science, Jinju, South Korea

**Keywords:** Tumour biomarkers, Prognostic markers

## Abstract

Rab27 is an essential molecule of vesicle fusion and trafficking in exosome secretion process, which plays important roles in cancer progression and metastasis. Recent studies reported that Rab27 expression is also associated with cancer prognosis. Therefore, we performed a meta-analysis to reveal the prognostic significance of Rab27 expression in solid cancer. Data were extracted by searching on PubMed, Embase and Cochrane library until February 15 2020. Pooled hazard ratio (HR) with confidence interval (CI) was calculated to evaluate the association between Rab27 expression and survival in solid cancer. Ten studies with 1434 cancer patients were including for this meta-analysis. High expression of Rab27 was associated with poor survival (HR 2.67, 95% CI 1.52–4.69, *p* = 0.001). High expression of Rab27A was significantly associated with lymph node metastasis (HR 1.53, 95% CI 1.00–2.34, *p* = 0.048). High expression of Rab27B was significantly correlated with lymph node and distant metastasis (HR 2.15, 95% CI 1.56–2.95, *p* < 0.001; HR 6.80, 95% CI 3.12–14.85, *p* < 0.001), and higher TNM stage (HR 2.55, 95% CI 1.78–3.65, *p* < 0.001). This meta-analysis revealed that Rab27 expression could be a potential prognostic marker in solid cancer.

## Introduction

Cancer is a common cause of morbidity and mortality throughout the world^1^. In 2018, more than 18 million new cancer patients were occurred and 9.5 million died^2^. In spite of desperate development of new remedies in recent years, the prognosis of cancer remains bleak^3^. Therefore, recognition of new biomarkers related to the progression of cancer is essential for improving clinical outcomes^4^.

Rab proteins are small GTPases consisting more than 70 members in human and work as regulators of proteins trafficking, membrane focusing and fusion, and vesicles transportation, which is one of the processes to control the functioning of cells, including cell proliferation, signal communication and protein transportation^5,6^.

Rab27 is one of the Rab proteins and is made of two components, Rab27A and Rab27B in vertebrates^6^. Rab27A and Rab27B are expressed in many kinds of secretory epithelial cells and are the essential substances of vesicle trafficking and fusion in the process of exosome secretion, which is known to play significant roles in the progression and metastasis of cancer by controlling the microenvironment of cancer^7–19^. Moreover, recent studies reported that Rab27A and Rab27B expression are related with the prognosis of cancer^8,20–28^.

However, the prognostic significance of Rab27 expression is not yet understand systematically in cancer. Therefore, we performed a comprehensive meta-analysis to estimate the prognostic significance of Rab27 expression in solid cancer.

## Results

### Study characteristics

**Figure 1:**
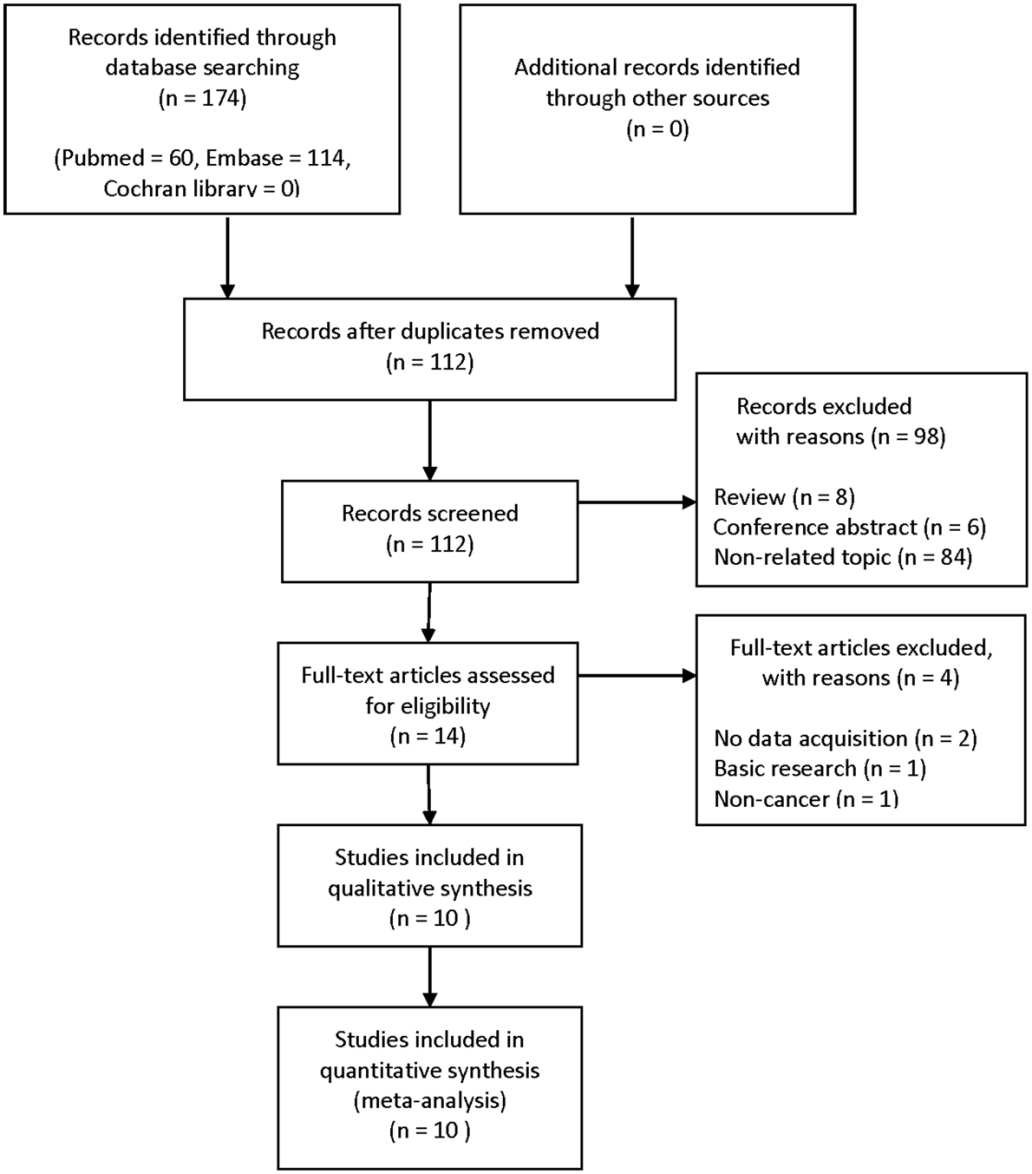
Flow diagram of study selection.

The literature selection flow of the included studies was presented in Fig. 1. Ten studies including 1434 patients were chosen for our meta-analysis. The basic characteristics of included studies were summarized in Table 1. All studies were published between 2012 and 2019 and were processed in Asia. The included studies were consisted of seven types of cancers, including renal cell carcinoma (n = 1), lung cancer (n = 2), ovarian cancer (n = 1), pancreatic cancer (n = 2), colorectal cancer (n = 2), breast cancer (n = 1), and hepatocellular carcinoma (n = 1). All studies performed immunohistochemistry to evaluate Rab27A or Rab27B expression in the human cancer tissue, and the majority of the cut-off value were scoring system using staining intensity and proportion. The Newcastle-Ottawa Scale (NOS) score is 7 to 8, which is considered to be high quality studies.

**Table 1:** Basic characteristics of the included studies.

Study	Country	Cancer type	Sample size	Gender (M/F)	Mean age (years)	Study period	Follow-up (months)	Survival outcome	Rab27 detection	Rab27 associated with prognosis	Cut-off value of Rab27 expression	Survival analysis	NOS
An et al.^23^	South Korea	Clear cell renal cell carcinoma	152	109/43	59.9 (32–83)	2000–2009	Mean 51.96	DSS	IHC	Rab27A	Moderate and strong intensity (> 1 +)	MVA	8
Koh et al.^26^	South Korea	Non-small cell lung cancer	133	111/22	Median 66 (31–77)	2002–2009	NA	DSS	IHC	Rab27B	> 30%, stronger than internal control	MVA	7
Zhang et al.^28^	China	Lung adenocarcinoma	80	44/36	NA	NA	NA	OS	IHC	Rab27B	High expression (≥ 3)	MVA	7
Ren et al.^20^	China	Ovarian cancer	103	0/103	NA	2004–2013	NA	OS	IHC	Rab27B	Staining scores with intensity and proportion (≥ 4.5)	MVA	7
Zhao et al.^8^	China	Pancreatic cancer	186	99/70	NA	2000–2010	NA	OS	IHC	Rab27B	Staining scores with intensity and proportion (≥ 91)	MVA	7
Shi et al.^22^	China	Colorectal carcinoma	112	73/39	65.14	2003–2008	NA	OS	IHC	Rab27A	Staining scores with intensity and proportion (≥ 4)	MVA	7
Wang et al.^26^	China	Pancreatic cancer	186	110/76	NA	2003–2010	NA	OS	IHC	Rab27A	Staining scores with intensity and proportion (≥ 91)	MVA	7
Bao et al.^24^	China	Colorectal cancer	113	73/40	65.2	2006–2008	NA	OS	IHC	Rab27B	Staining scores with intensity and proportion (≥ 4)	MVA	7
Zhang et al.^27^	China	Breast cancer	221	0/221	47	2000–2002	Median 79 (60–112)	DSS	IHC	Rab27B	Staining scores with intensity and proportion (> 3)	MVA	8
Dong et al.^21^	China	Hepatocellular carcinoma	148	108/35	51.6 (29–72)	2005–2009	NA	OS	IHC	Rab27B	Positive expression	MVA	7

*DSS* disease-specific survival, *IHC* immunohistochemistry, *MVA* multivariate analysis, *NA* not available, *NOS* Newcastle–Ottawa Scale, *OS* overall survival.

### Association between Rab27 expression and survival

**Figure 2:**
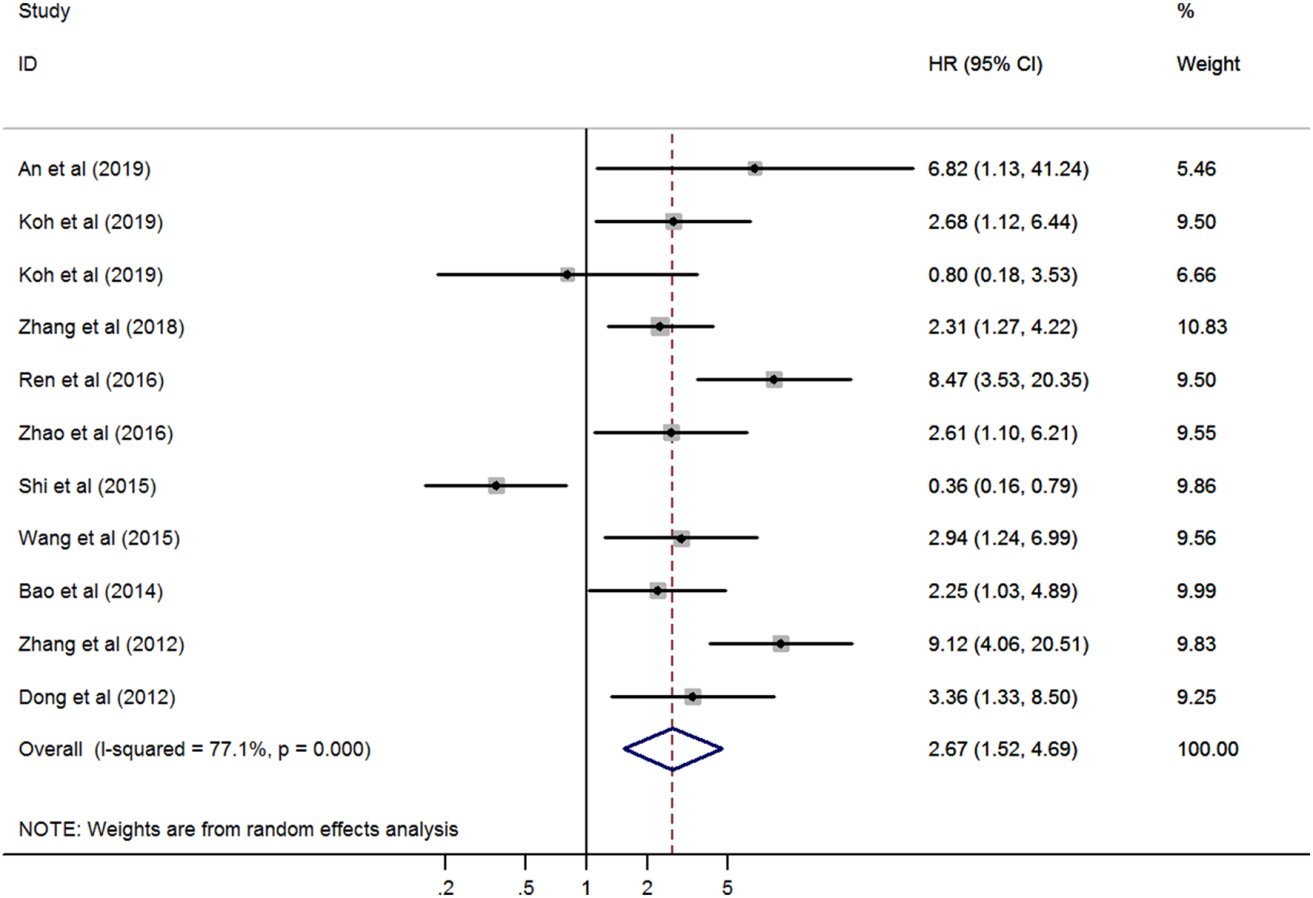
Forest plot of the association between Rab27 expression and survival.

Ten studies including 1434 cancer patients reported the association between Rab27 expression with survival. The pooled HR was evaluated using random-effects model. High expression of Rab27 was associated with poor survival (Hazard Ratio [HR] 2.67, 95% confidence interval [CI] 1.52–4.69, *p* = 0.001) although with heterogeneity (I^2^ = 77.1%, *p* < 0.001) (Fig. 2).

**Figure 3:**
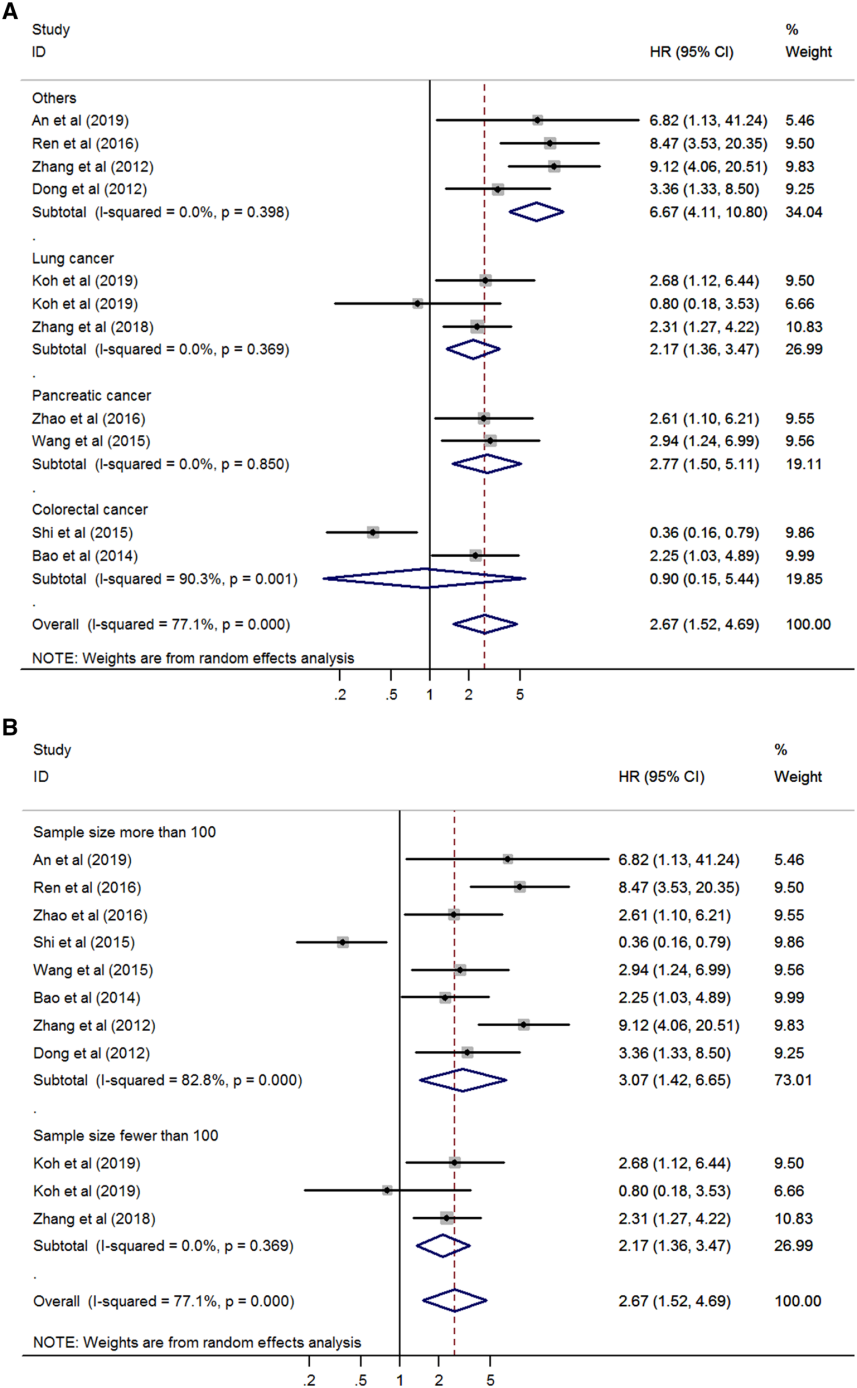

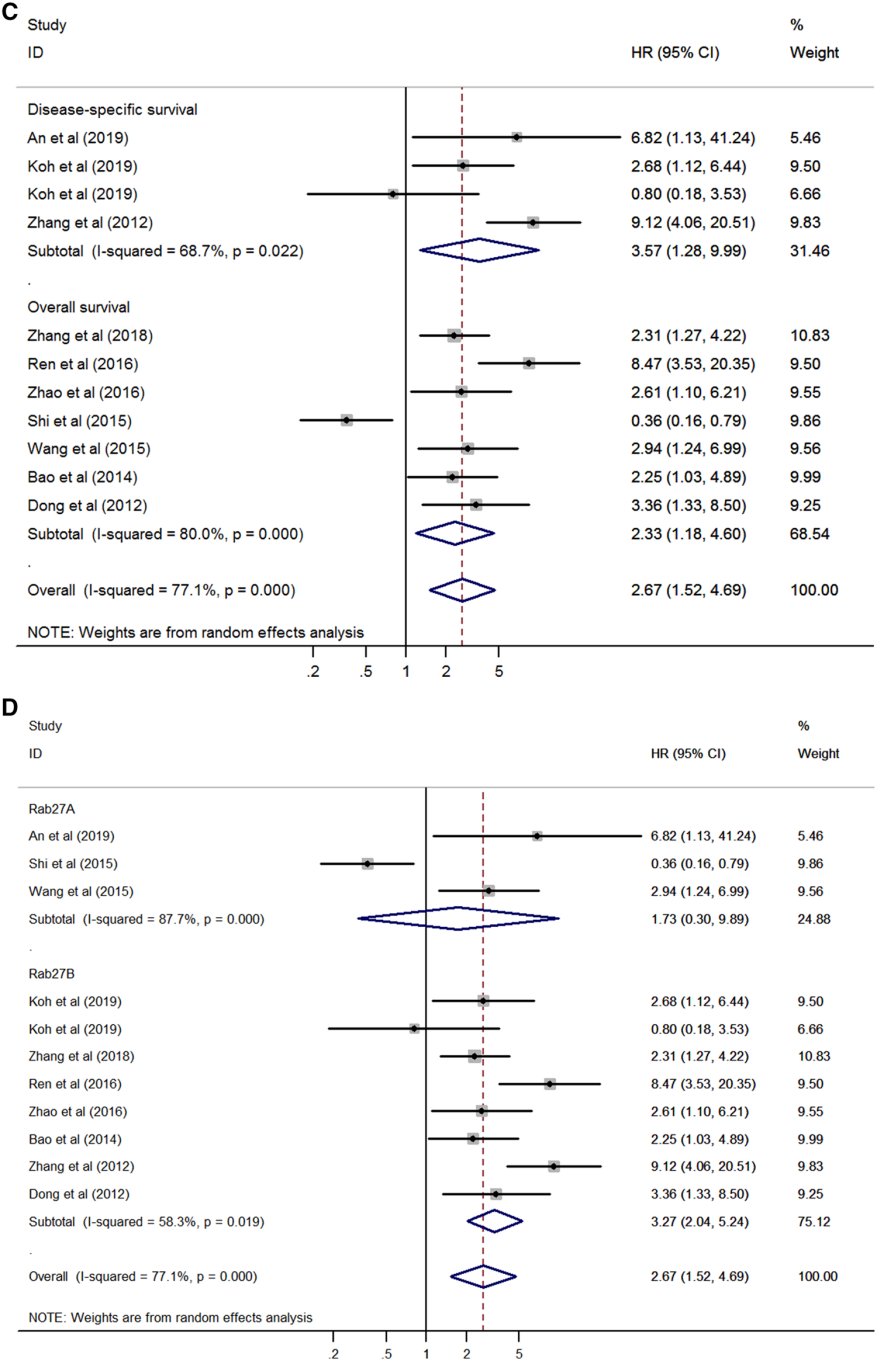
Forest plot for survival stratified by cancer type (**A**), sample size (**B**) survival outcome (**C**), and protein type (**D**).

Subgroup analysis was performed to investigate the potential source of heterogeneity and revealed that cancer type, sample size, survival outcomes, and protein type could be the main sources of heterogeneity (Table 2). According to the stratification by cancer type, the result of poor survival in patients with high expression of Rab27 was consistently identified in lung cancer (HR 2.17, 95% CI 1.36–3.47, *p* = 0.001) and pancreatic cancer (HR 2.77, 95% CI 1.50–5.11, *p* = 0.001) except colorectal cancer (HR 0.90, 95% CI 0.15–5.44, *p* = 0.911) (Fig. 3A). Based on the sample size, poor survival was correlated to Rab27 expression in both groups (sample size more than 100, HR 3.07, 95% CI 1.42–6.65, *p* = 0.004; sample size fewer than 100, HR 2.17, 95% CI 1.36–3.47, *p* = 0.001) (Fig. 3B). On the analysis of survival outcomes, there was significant relationship between Rab27 expression and poor survival in both groups (disease-specific survival, HR 3.57, 95% CI 1.28–9.99, *p* = 0.015; overall survival, HR 2.33, 95% CI 1.18–4.60, *p* = 0.015) (Fig. 3C). When it comes to the protein type, there was a significant result in Rab27B (HR 3.27, 95% CI 2.04–5.24, *p* < 0.001), but not in Rab27A (HR 1.73, 95% CI 0.30–9.89, *p* = 0.538) (Fig. 3D).

**Table 2:** Subgroup analysis of the association between Rab27 expression and survival.

Subgroup	Number of studies	Number of patients	Pooled HR (95% CI)	*p* value	Heterogeneity	
					I^2^ (%)	*p* value
**Cancer type**						
Colorectal cancer	2	225	0.90 (0.15–5.44)	0.911	90.3	0.001
Lung cancer	2	213	2.17 (1.36–3.47)	0.001	0.0	0.369
Pancreatic cancer	2	372	2.77 (1.50–5.11)	0.001	0.0	0.850
Others	4	624	6.67 (4.11–10.80)	< 0.001	0.0	0.398
**Sample size**						
Fewer than 100	2	213	2.17 (1.36–3.47)	0.001	0.0	0.369
More than 100	8	1221	3.07 (1.42–6.65)	0.004	82.8	< 0.001
**Survival outcome**						
DSS	3	506	3.57 (1.28–9.99)	0.015	68.7	0.022
OS	7	928	2.33 (1.18–4.60)	0.015	80.0	< 0.001
**Protein type**						
Rab27A	3	450	1.73 (0.30–9.89)	0.538	87.7	< 0.001
Rab27B	7	984	3.27 (2.04–5.24)	< 0.001	58.3	0.019

*CI* confidence interval, *DSS* disease-specific survival, *HR* hazard ratio, *OS* overall survival.

### Association between Rab27 expression and clinicopathological characteristics

**Figure 4:**
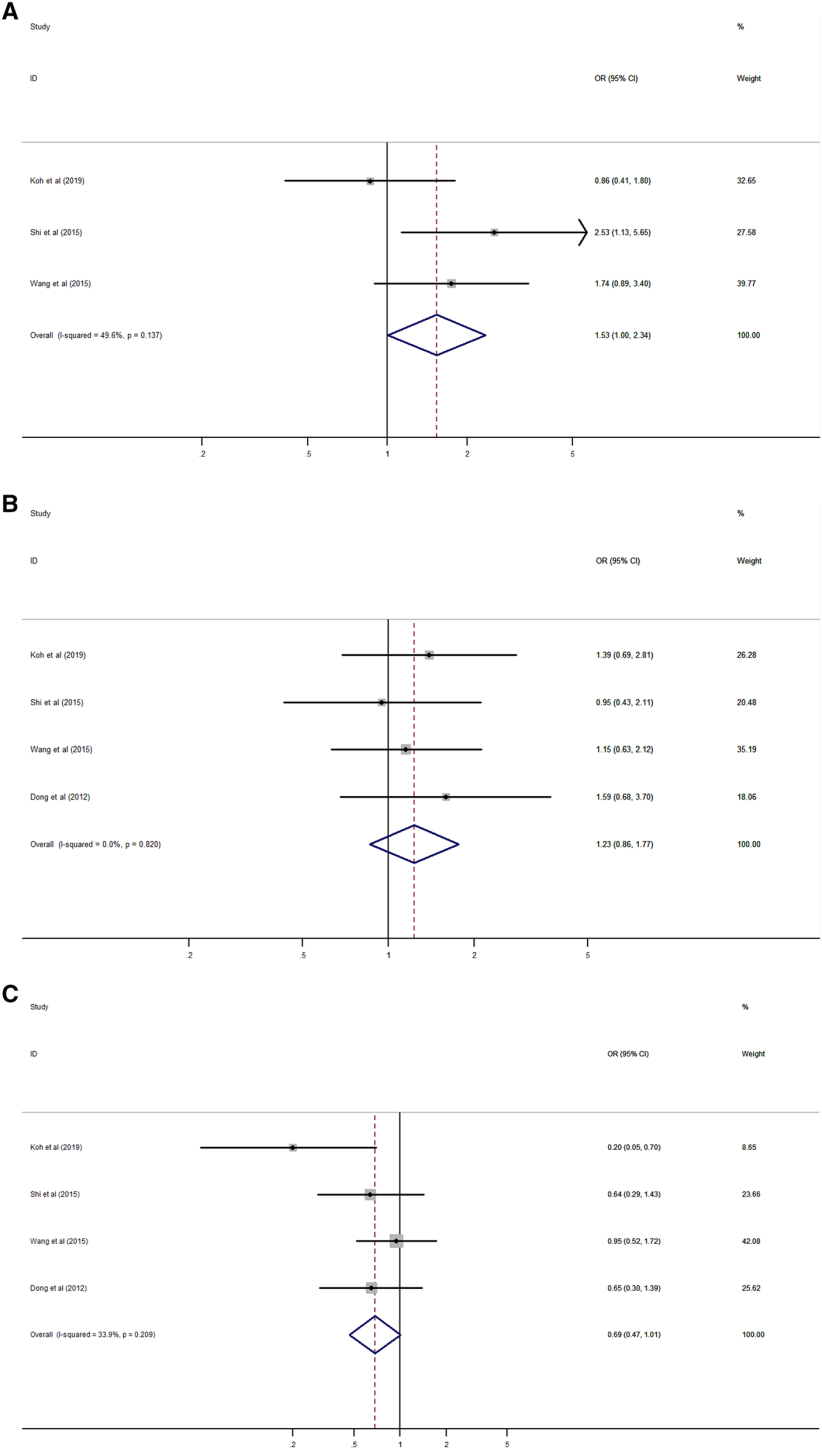

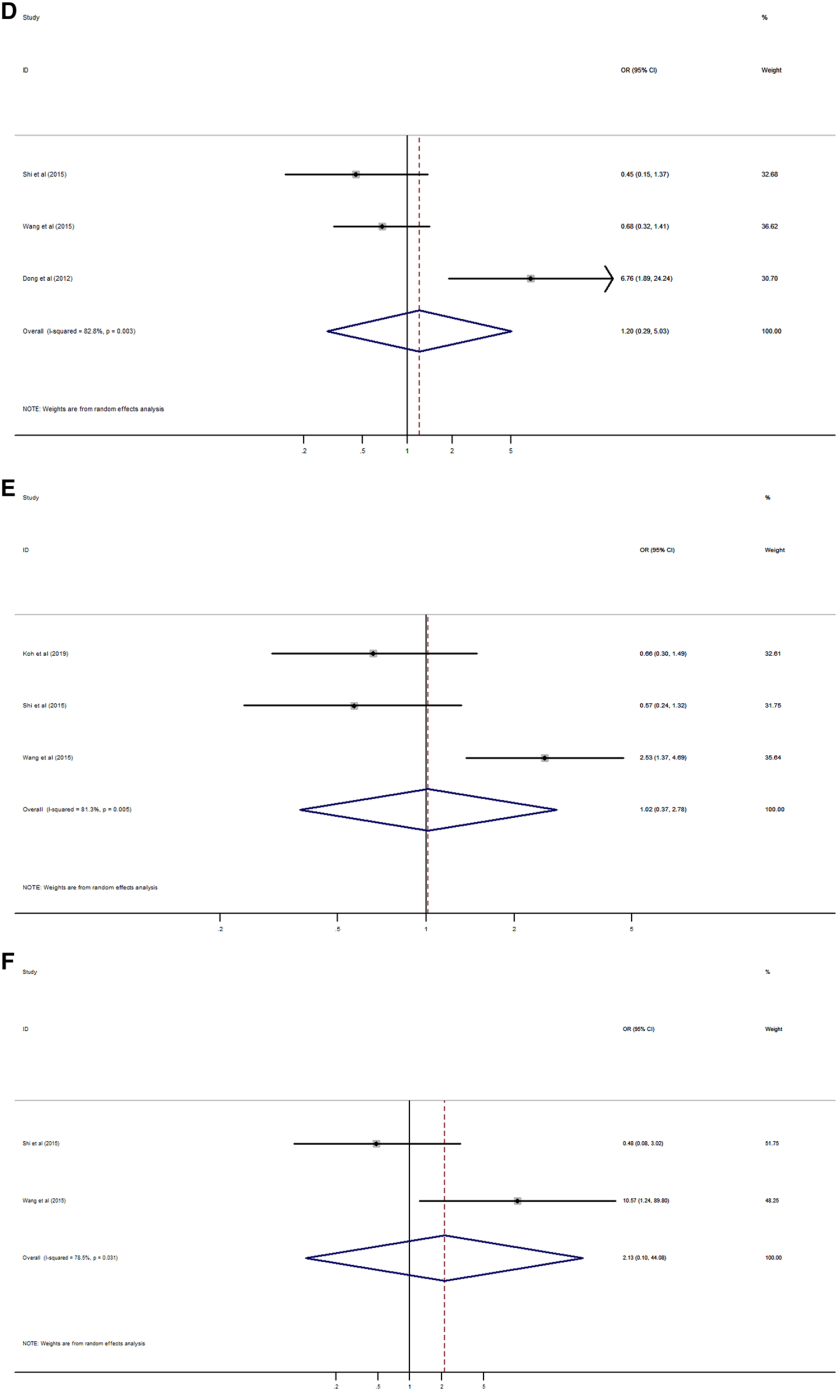

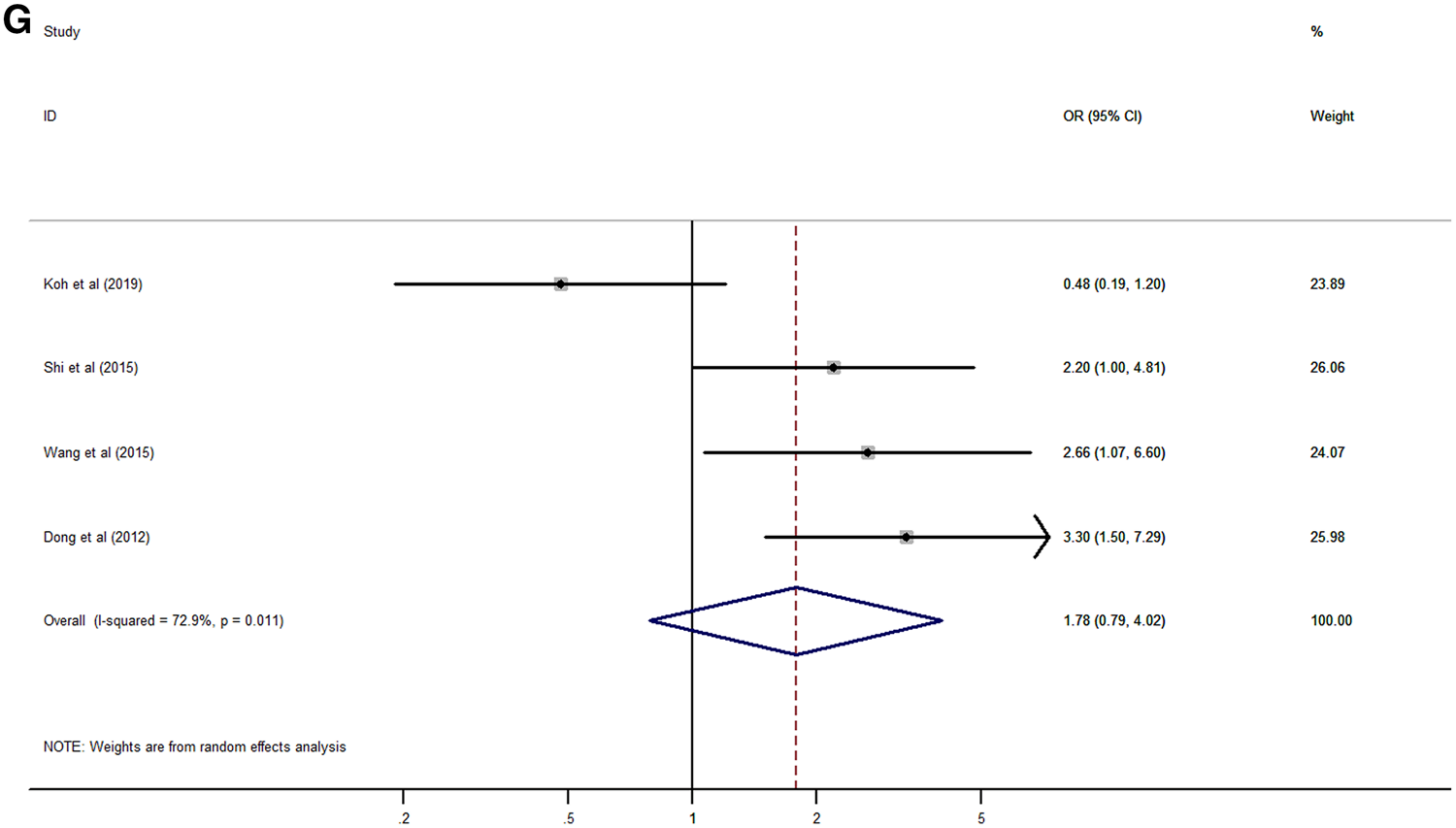
Forest plot of the association between Rab27A expression and clinicopathological characteristics. (**A**) lymph node metastasis, (**B**) age, (**C**) gender, (**D**) tumor grade, (**E**) tumor stage, (**F**) distant metastasis, (**G**) TNM stage.

Analysis of the association between Rab27 expression and clinicopathological characteristics of cancer patients was summarized in Table 3. The results suggested that high expression of Rab27A was significantly associated with lymph node metastasis (HR 1.53, 95% CI 1.00–2.34, *p* = 0.048) (Fig. 4A). However, there was no significant relationship between Rab27A expression with age, gender, tumor grade and stage, distant metastasis, and TNM stage (Fig. 4B–G).

**Table 3:** Association between Rab27 expression and clinicopathological characteristics.

Characteristic	Number of studies	Number of patients	Pooled OR (95% CI)	*p* value	Heterogeneity		
					I^2^ (%)	*p* value	Model
**Age (old vs young)**							
Rab27A	4	579	1.23 (0.86–1.77)	0.255	0.0	0.820	Fixed
Rab27B	7	984	1.11 (0.84–1.48)	0.450	32.4	0.181	Fixed
**Gender (male vs female)**							
Rab27A	4	579	0.69 (0.47–1.01)	0.057	33.9	0.209	Fixed
Rab27B	5	660	0.88 (0.43–1.82)	0.729	74.2	0.004	Random
**Tumor size (large vs small)**							
Rab27B	3	414	1.17 (0.77–1.79)	0.454	0.0	0.386	Fixed
**Tumor grade (high vs low)**							
Rab27A	3	446	1.20 (0.29–5.03)	0.800	82.8	0.003	Random
Rab27B	6	851	1.33 (0.75–2.35)	0.333	55.8	0.046	Random
**Tumor stage (high vs low)**							
Rab27A	3	431	1.02 (0.37–2.78)	0.974	81.3	0.005	Random
Rab27B	5	723	1.43 (1.80–2.57)	0.229	61.3	0.036	Random
**Lymph node metastasis (present vs absent)**							
Rab27A	3	431	1.53 (1.00–2.34)	0.048	49.6	0.137	Fixed
Rab27B	6	836	2.15 (1.56–2.95)	< 0.001	27.9	0.225	Fixed
**Distant metastasis (present vs absent)**							
Rab27A	2	298	2.13 (0.10–44.08)	0.624	78.5	0.031	Random
Rab27B	3	422	6.80 (3.12–14.85)	< 0.001	0.0	0.982	Fixed
**TNM stage (high vs low)**							
Rab27A	4	579	1.78 (0.79–4.02)	0.167	72.9	0.011	Random
Rab27B	6	881	2.55 (1.78–3.65)	< 0.001	30.9	0.204	Fixed

*CI* confidential interval, *OR* odds ratio, *TNM* tumor-node-metastasis.

**Figure 5:**
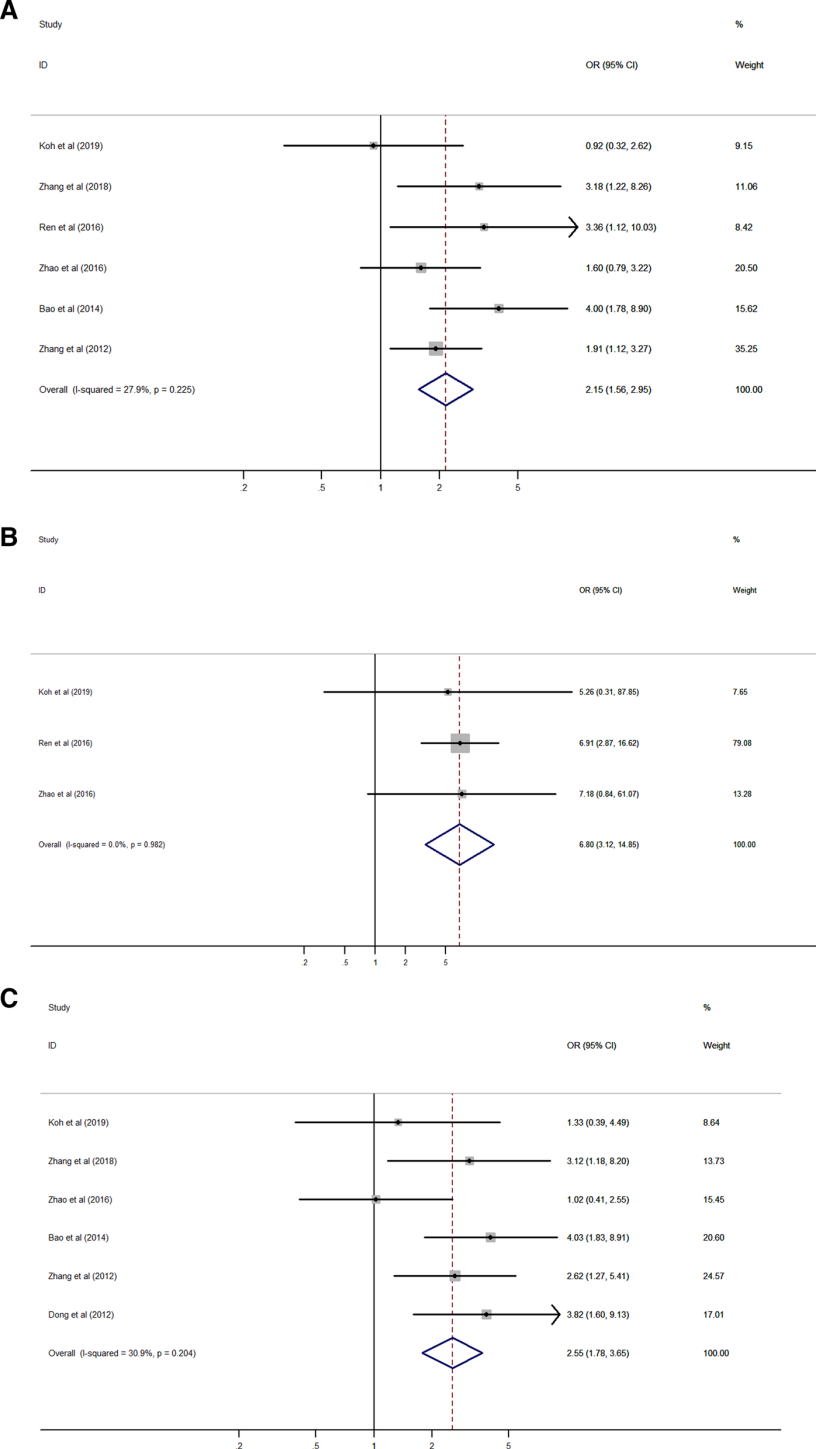

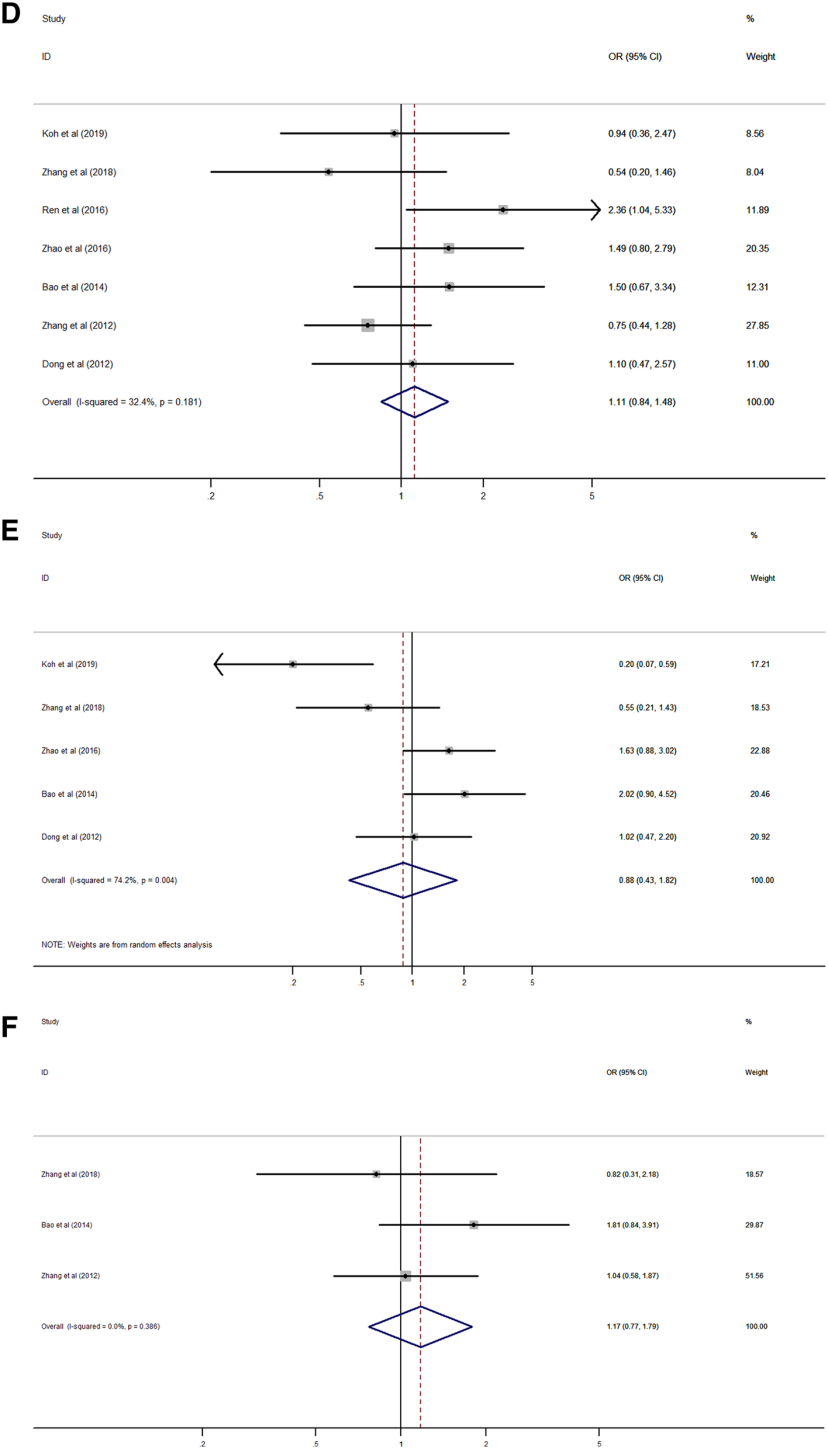

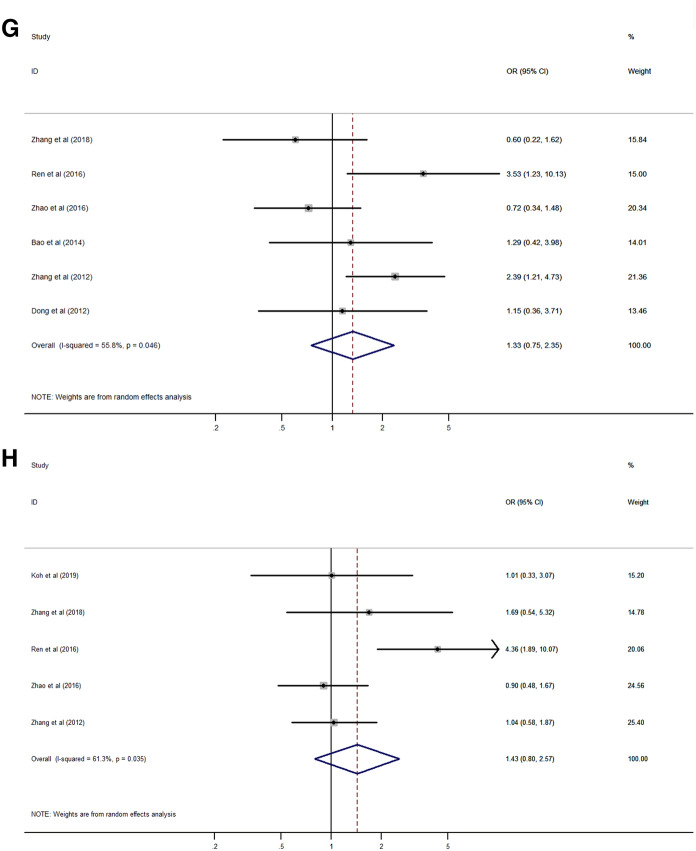
Forest plot of the association between Rab27B expression and clinicopathological characteristics. (**A**) lymph node metastasis, (**B**) distant metastasis, (**C**) TNM stage, (**D**) age, (**E**) gender, (**F**) tumor size, (**G**) tumor grade, (**H**) tumor stage.

High expression of Rab27B was significantly correlated with lymph node and distant metastasis (HR 2.15, 95% CI 1.56–2.95, *p* < 0.001; HR 6.80, 95% CI 3.12–14.85, *p* < 0.001) (Fig. 5A,B), and higher TNM stage (HR 2.55, 95% CI 1.78–3.65, *p* < 0.001) (Fig. 5C). But, there was no significant association between Rab27B expression with age, gender, tumor size, grade and stage (Fig. 5D–H).

### Publication bias and sensitivity analysis

**Figure 6:**
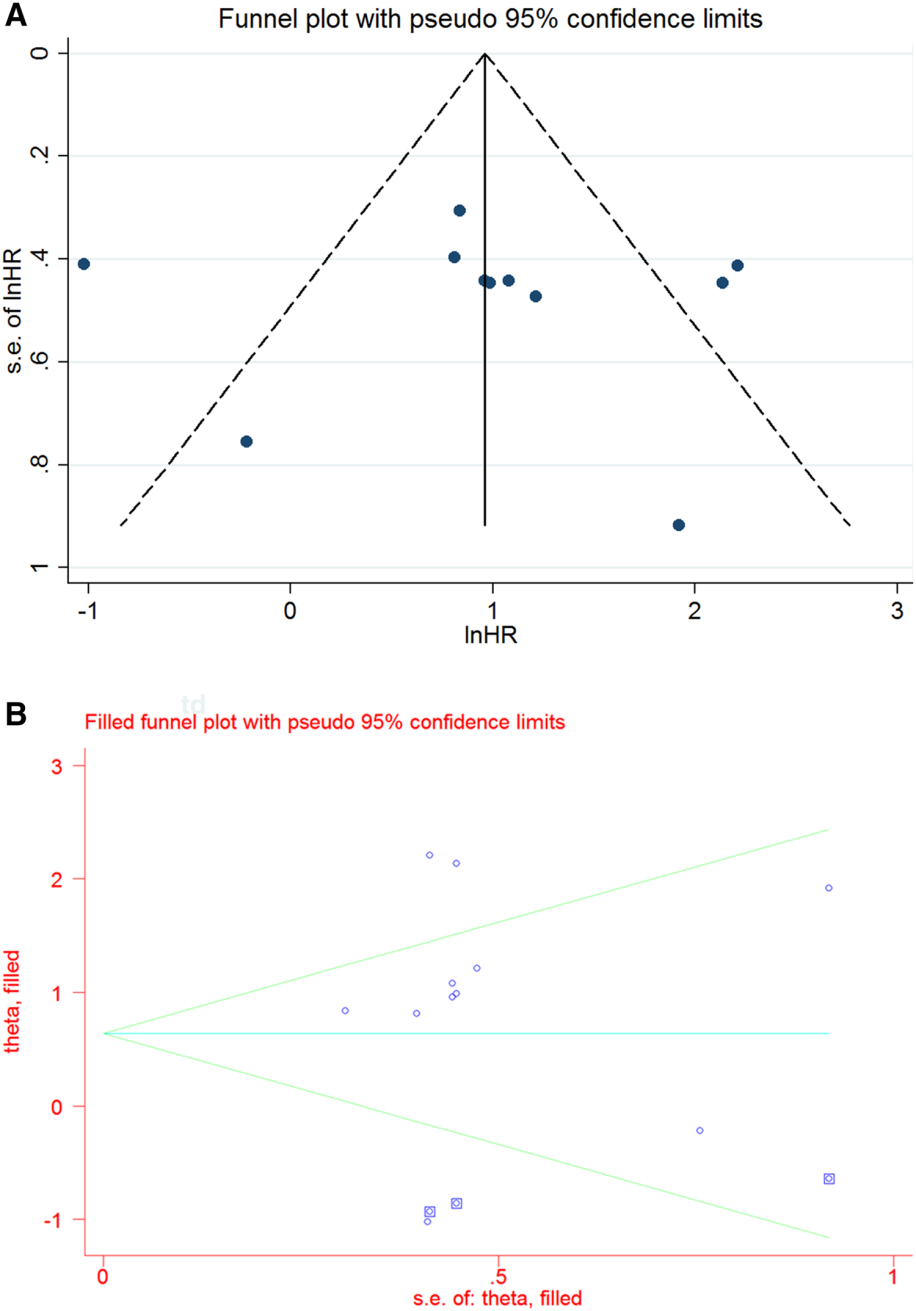
Funnel plot (**A**) and trim and fill method (**B**) of the association between Rab27 expression and survival.

Visual inspection of the Funnel plot revealed asymmetry (Fig. 6A). This suggests the possibility of a visual bias in publishing, however the Egger’s test was not statistically significant (*p* = 0.849). Thus, the trim and fill method was applied. The results showed the significant association between Rab27 expression with survival (HR 1.85, 95% CI 1.02–3.36, *p* = 0.042) (Fig. 6B).

**Figure 7:**
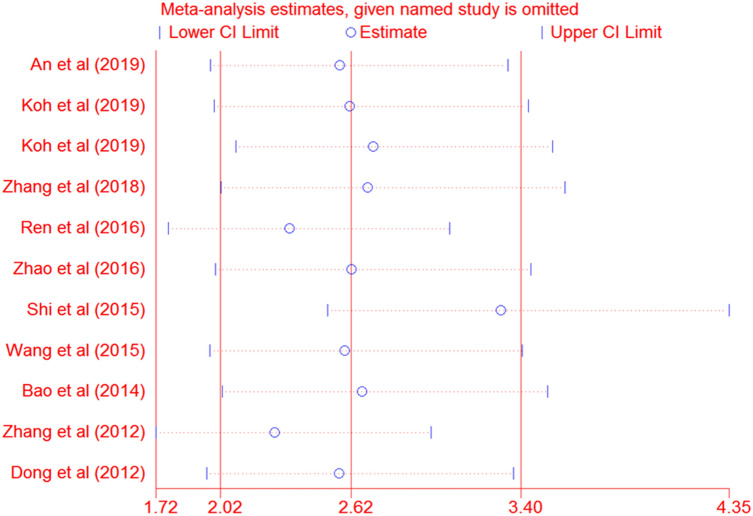
Sensitivity analysis of the association between Rab27 expression and survival.

The sensitivity analysis was conducted to verify the reliability of our results. The pooled HR for survival was not influenced after one by one, excluding each study, indicating our results are consistent and credible (HR 2.62, 95% CI 2.02–3.40) (Fig. 7).

## Discussion

The Rab family members are key regulators that involve in the replacement of substances in the various stages of vesicular trafficking^17^. Rab27 is a valuable member in Rab family and contains Rab27A and Rab27B, which share 70% sequence identity of the amino acid level^17,22^. Rab27 is located on the membrane-bound compartments and takes part in the docking multivesicular endosomes, which is known to control exosome secretion^17,29,30^. Recent studies have demonstrated that Rab27 regulates exosome secretion in the many kinds of cells, including dendritic cell, cervical cancer cells, breast cancer cells, melanoma cells, bladder cancer cells, and lung cancer cells^6^. Moreover, Rab27 has been revealed to function a critical role in the proliferation and invasion of cancer cells by the controlling of exosome secretion, which modulates the tumor microenvironment and the function of cancer cell^6^. Guo et al. showed that Rab27A affects melanoma cell proliferation and motility, and promotes melanoma invasion and metastasis by mediating exosomes^13–16^. Akavia et al. also reported that Rab27A contributes to proliferation in melanoma by the regulation of vesicular trafficking^11^. Liu et al. and Li et al. revealed that Rab27 plays significant roles in cell proliferation and invasion in bladder cancer and pancreatic cancer cells, respectively^17,18^. Bobrie et al. and Peinado et al. demonstrated that Rab27A can enhance tumor progression and metastasis by the modification of exosome secretion and tumor microenvironment^12,19^. Furthermore, some studies have reported that Rab27 expression is associated with the prognosis of cancer^8,20–28^. Thus, we evaluated that the association between Rab27A expression and survival.

This meta-analysis of 10 studies including 1434 patients assessing the prognostic significance of Rab27 expression in solid cancer revealed that high expression of Rab27 was a potential prognostic marker for poor survival in cancer patients.

In the subgroup analysis of cancer type, poor survival was related to Rab27 expression in lung cancer and pancreatic cancer without heterogeneity. On the other hand, survival was not significantly correlated with Rab27 expression in colorectal cancer. This seems to be due to the conflicting results of the two studies in this group. Thus, additional research is needed in colorectal cancer. Regarding the subgroup of sample size and survival outcomes, poor survival was correlated to Rab27 expression in both groups, respectively. As showed in Fig. 3D, poor survival was significantly associated with high expression of Rab27B, but not with Rab27A. This seems to be due to the study of Shi et al. that reported high expression of Rab27A indicates favorable prognosis showing contrary results with other studies.

With regard to the clinicopathological characteristics, our analysis revealed that high expression of Rab27 was significantly correlated with lymph node metastasis. Moreover, Rab27B was also significantly associated with distant metastasis and TNM stage. This results strongly suggests that high expression of Rab27 is related to the prognosis of cancer patients.

This meta-analysis has the following limitations. First, all included studies originated from Asia, which may lead to our results not reflecting western countries. Second, cancer type and sample size were small, undermining the credibility of our results. Third, the discrepancy of cut-off value determining Rab27A and Rab27B expression may affect our results. However, we demonstrated a comprehensive analysis of the association between Rab27A and Rab27B expression with survival in solid cancer. To the best of our knowledge, this meta-analysis is the first report to systematically evaluate the association between Rab27 expression with survival in solid cancer.

In conclusion, this systematic review and meta-analysis revealed that Rab27 expression could be a prognostic marker in solid cancer.

## Methods

### Search strategy and selection criteria

We searched the PubMed, Embase and Cochrane library until February 15 2020 using the following terms: (Rab27A or Rab27B) and (cancer or tumor or carcinoma or neoplasm or malignancy) and (prognostic or predict or prognosis or survival or outcome). We reviewed all significant publications in the references of the articles.

The inclusion criteria were: (1) Rab27A or Rab27B was detected in the human cancer tissue, which diagnosed by pathologic examination; (2) the association between Rab27A or Rab27B and survival outcomes was reported; (3) the HR and 95% CI for survival outcomes were provided.

The exclusion criteria were: (1) duplicate studies; (2) conference abstracts, case reports, reviews, letters, and non-English articles; (3) preclinical studies, such as laboratorial or in vitro studies.

### Data extraction

Two authors independently collected the following data from the included studies: first author, year of publication, country, cancer type, sample size, clinicopathological characteristics, study period, follow-up period, survival outcomes, Rab27 associated with prognosis, cut-off value of Rab27 expression, and survival data. When there was a disagreement, we reached an agreement through discussion.

### Quality assessment

Two authors independently evaluated the quality of included studies by the NOS. The NOS scores ranged from 0 to 9. The studies with NOS scores of greater than 6 were regarded as a high quality.

### Statistical analysis

Meta-analysis was performed using StataSE12 (Stata, College Station, TX, USA). Cochran’s Q and I^2^ statistics were used to assess the heterogeneity among the included studies. An I^2^ > 50% or a *p* value < 0.1 was considered as statistically significant in a random-effects model. Pooled HR and 95% CI were calculated for evaluating the prognostic significance of Rab27 expression. Subgroup analysis was performed to assess the source of heterogeneity. Funnel plot and Egger’s test were also performed to evaluate the publication bias. And the sensitivity analysis was applied to assess the reliability of the pooled results. A *p* value < 0.05 was regarded as statistically significant.
